# ECG Electrode Placements for Magnetohydrodynamic Voltage Suppression and improving Cardiac Gating in high-field MRI

**DOI:** 10.1186/1532-429X-18-S1-P328

**Published:** 2016-01-27

**Authors:** Thomas S Gregory, John Oshinski, Ehud J Schmidt, Zion T Tse

**Affiliations:** 1 grid.213876.9000000041936738XCollege of Engineering, University of Georgia, Athens, GA USA; 2 grid.412162.20000000404415844Radiology, Emory University Hospital, Atlanta, GA USA; 3 grid.62560.370000000403788294Radiology, Brigham and Women's Hospital, Boston, MA USA

## Background

The accuracy of Electrocardiogram (ECG) gating for synchronization of MR scanner image acquisition and cardiac electrical activity is of great importance for acquiring high-quality Cardiac Magnetic Resonance (CMR) images free of motion artefacts. The distortion of ECG traces by Magnetohydrodynamic Voltages (VMHD) induced by interaction between the MRI static magnetic field (B_0_) and rapid left-ventricular blood ejection during systole can lead to false and/or intermittent QRS complex detection and images with severe motion artefacts [1]. We hypothesized that an optimized electrode placement for the reduction of induced VMHD could be derived based on a thoracic model to increase the accuracy of QRS complex detection.

## Methods

**Figure 1:**
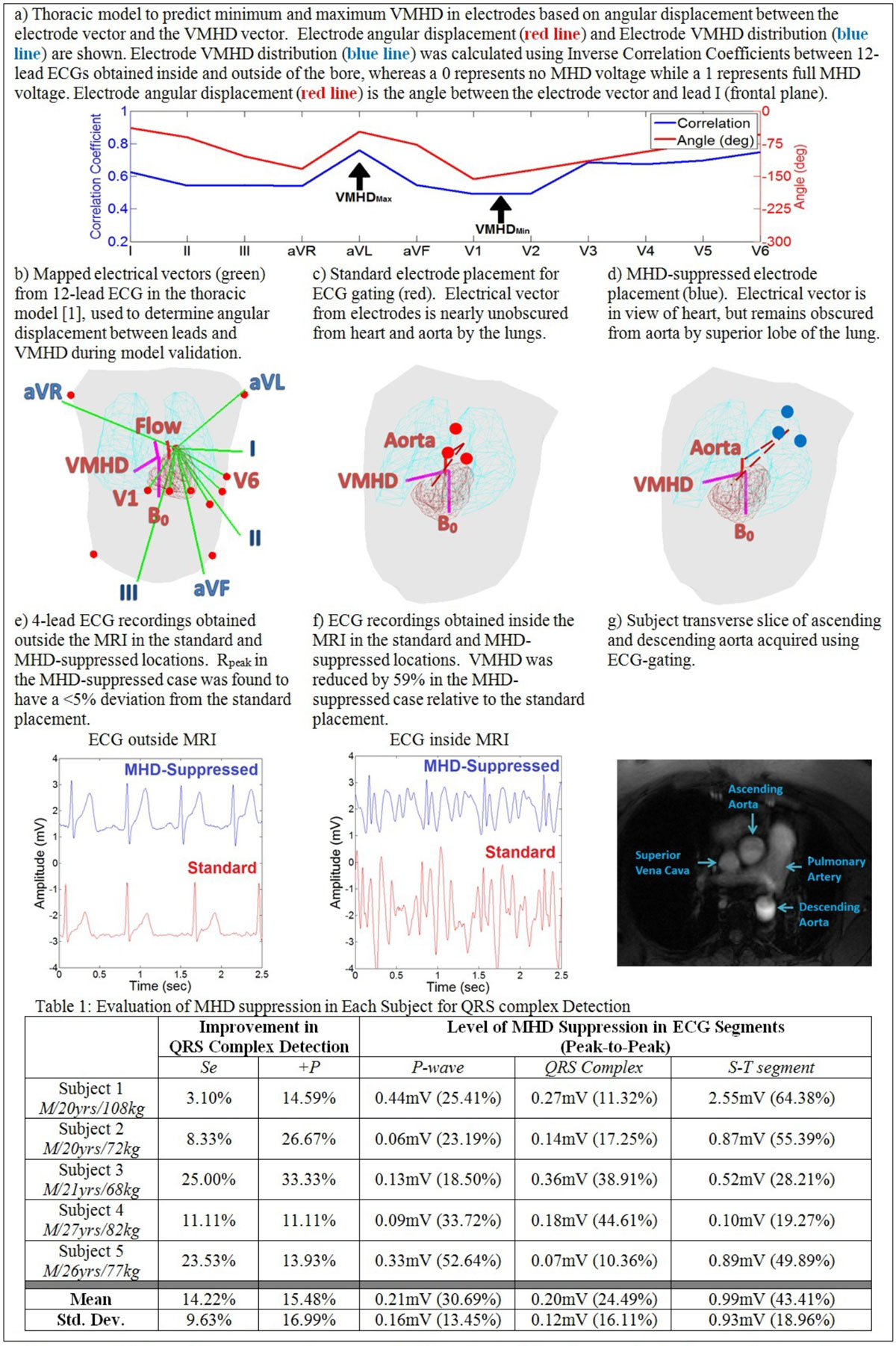
**Development of electrode placement recommendations for increased accuracy in QRS complex detection and MRI gating**.

A vector model based on thoracic geometry [2] was calibrated using 12-lead ECGs recorded in four subjects in a GE 3T scanner to estimate VMHD distributions on the thorax. 4-lead ECG electrode placement was then optimized to: (1) minimize VMHD magnitude and (2) reduce displacement from the SA node for maximizing QRS complex amplitude (Figure 1a,b). A gradient-descent optimization routine was utilized to predict the optimal 4-lead ECG placement based on angular displacement and heart/aorta geometry (Figure 1c,d). Model results were then validated using five healthy subjects. Sensitivity (Se) and Positive Predictability (+P) rates for detection of R-waves were compared between conventional and MHD-suppressed lead placements for single-lead QRS complex detection [3].

## Results

A 43.41% reduction in VMHD during the S-T segment (Figure 1f) was observed in ECGs using the MHD-suppressed placement relative to the conventional placement, while preserving the QRS complex (Figure 1e), resulting in an average increase in the Se and +P rate of 14.22% and 15.48%, respectively (Figure 1e-g). R_peak_ amplitude inside the MRI in the MHD-suppressed placement had <5% deviation from the standard placement outside of the MRI (Figure 1e). As compared to the conventional electrode placement (Figure 1c-d), MHD suppression may result from decreased visibility of the aorta through the lungs at the MHD-suppressed placement.

## Conclusions

Electrode placement recommendations were computed and validated in a 3T MRI, illustrating an increased accuracy in QRS complex detection using the MHD-suppressed placement.
